# GNSS/Electronic Compass/Road Segment Information Fusion for Vehicle-to-Vehicle Collision Avoidance Application

**DOI:** 10.3390/s17122724

**Published:** 2017-11-25

**Authors:** Rui Sun, Qi Cheng, Dabin Xue, Guanyu Wang, Washington Yotto Ochieng

**Affiliations:** 1 College of Civil Aviation, Nanjing University of Aeronautics and Astronautics, Nanjing 211106, China; qi_cheng@outlook.com (Q.C.); xdb@nuaa.edu.cn (D.X.); guanyu_wang@outlook.com (G.W.); w.ochieng@imperial.ac.uk (W.Y.O.); 2 State Key Laboratory of Geo-Information Engineering, Xi’an 710054, China; 3 Centre for Transport Studies, Imperial College London, London SW7 2AZ, UK

**Keywords:** ITS, GNSS, autoregressive motion model, particle filter, collision avoidance

## Abstract

The increasing number of vehicles in modern cities brings the problem of increasing crashes. One of the applications or services of Intelligent Transportation Systems (ITS) conceived to improve safety and reduce congestion is collision avoidance. This safety critical application requires sub-meter level vehicle state estimation accuracy with very high integrity, continuity and availability, to detect an impending collision and issue a warning or intervene in the case that the warning is not heeded. Because of the challenging city environment, to date there is no approved method capable of delivering this high level of performance in vehicle state estimation. In particular, the current Global Navigation Satellite System (GNSS) based collision avoidance systems have the major limitation that the real-time accuracy of dynamic state estimation deteriorates during abrupt acceleration and deceleration situations, compromising the integrity of collision avoidance. Therefore, to provide the Required Navigation Performance (RNP) for collision avoidance, this paper proposes a novel Particle Filter (PF) based model for the integration or fusion of real-time kinematic (RTK) GNSS position solutions with electronic compass and road segment data used in conjunction with an Autoregressive (AR) motion model. The real-time vehicle state estimates are used together with distance based collision avoidance algorithms to predict potential collisions. The algorithms are tested by simulation and in the field representing a low density urban environment. The results show that the proposed algorithm meets the horizontal positioning accuracy requirement for collision avoidance and is superior to positioning accuracy of GNSS only, traditional Constant Velocity (CV) and Constant Acceleration (CA) based motion models, with a significant improvement in the prediction accuracy of potential collision.

## 1. Introduction

With increasing traffic in cities, crashes are becoming a major safety concern. In particular, according to the U.S. National Highway Traffic Safety Administration (NHTSA), car accidents dominate crashes in cities, occurring every minute of the day [1]. It has been argued that the development and implementation of early warning systems could have the impact of reducing crashes. This could be achieved through the collision avoidance application or service of Intelligent Transport Systems (ITS). 

Research to date has explored a number of aspects of collision detection. Araki et al. [2,3] developed a collision-avoidance system based on an on-board laser radar and a Charge Coupled Device (CCD) camera and applied fuzzy logic to evaluate the potential for a collision using the relative distance, velocity and acceleration of both vehicles. However, the sensors are weather sensitive and do not support collision detection in all directions. The former also results in reduced system performance. In addition, the work is preliminary without quantified field test results in terms of collision detection accuracy. Risack et al. [4] developed a lane-keeping assistant video-based system with the capability for collision avoidance by using vehicle position and the time to cross a lane. By evaluating different lane departure detection algorithms, the blinker and braking state as well as steering activity, an acoustic signal is transmitted to the driver to warn of an impending collision. However, the performance of the video-based system is also affected by the weather. Furthermore, no field test results have been reported. Ujjainiya and Chakravarthi [5] proposed a cost-effective vehicle collision avoidance system based on vision sensors and image processing algorithms. Although it is argued that the model could effectively detect the vehicle edge, sensitivity to the weather is still a major issue, in addition to a lack of evidence on the performance of the system. Ueki et al. [6] developed a collision avoidance system by inter-vehicle communication technology. This research focused on the vehicle communication network, without addressing the quality of the real-time states of vehicles, which are critical inputs for the detection of collision. 

Ferrara and Paderno [7] investigated the possibility of designing a driver assistance system for cars capable of making a decision between an emergency braking and a collision avoidance manoeuvre and generating an appropriate automatic action, as long as a collision is likely to occur within 1 s. This research mainly focussed on the control mechanism for intervention, assuming that the vehicle state is error free, which is not realistic. Huang and Tan [8] discussed error propagation and robustness of a cooperative collision warning system with an Extended Kalman Filter (EKF) based trajectory prediction algorithm for vehicles equipped with a Differential Global Positioning System (DGPS). This paper only analysed the effect of loss and latency/delay issues related to communication on collision avoidance. The quality of vehicle state parameters was not addressed. In addition, the traditional Constant Velocity (CV) model was with the EKF based trajectory prediction. The CV model does not account for changes in vehicle motion. Ong and Lachapelle [9] proposed a Global Navigation Satellite System (GNSS) based vehicle-pedestrian and vehicle-cyclist crash avoidance system. They analyzed the effectiveness of different types of GNSS, including real-time kinematic (RTK) GNSS, DGPS and single point positioning based Global Positioning System (GPS). The test results showed that it is feasible to use GNSS for collision avoidance. Although the work addressed vehicle/pedestrian collision avoidance, the idea is transferable to and adaptable for vehicle-to-vehicle (V2V) collision avoidance. The research also indicated that GNSS only based method is not reliable enough for collison avoidance and recommended its integration with other sensors. Toledo-Moreo and Zamora-Izquierdo [10] developed a GPS/IMU/spatial data integrated lateral and longitudinal information supported collision avoidance system. In this system, the vehicle state prediction is realized by a Bi-Dimensional Interactive Multiple Model (2D-IMM) filter in which longitudinal and lateral motions of the vehicle are distinguished and different maneuvering described by different kinematic models. It is argued that the designed algorithm is effective at detecting the manouvers of the vehicles under the designed scenarios by choosing an appropriate motion model. However, model mismatch is a major limitation. 

From the literature above, weather sensitivity and the lack of detection in all directions are critical limitations for vision and radar based collision detection methods [2,3,4,5]. On the other hand, advanced RTK GNSS based technology, in principle capable of sub-meter positioning accuracy, augmented with data from other sensors to provide higher integrity, continuity and availability, has the potential to provide vehicle states estimation to support high performance collision avoidance in all weather conditions and in all directions. Through appropriate communication between vehicles equipped with GNSS-based sensors, position and real-time dynamic information of the relevant vehicles can be used for the detection of an impending collision and avoidance measures evoked. However, for collision detection, there are specific scenarios that require the real-time accuracy of dynamic state estimation to be improved [9]. In particular, state estimation accuracy deteriorates during abrupt acceleration and decelerations situations, which partly is the result of the current vehicle motion model limitations [6,7,8,10]. 

In summary, in order to address the limitations of weather sensitivity, lack of multi-directional detection, motion model limitations and low performance of the current collision detection approaches and provide the required level of performance for state estimation in terms of accuracy, integrity, continuity and availability, this paper develops a novel Particle Filter (PF) Autoregressive (AR) model based GPS/electronic compass/road segment data fusion algorithm for V2V collision avoidance. The integration of GNSS with electronic compass and road segment data provides higher performance vehicle state estimation, which is weather insensitive and could support collision detection in all directions. The AR based motion model is adaptive based on historical motion states and therefore addresses the limitations of the traditional Constant Velocity (CV) and Constant Acceleration (CA) based motion models. 

The contributions of the paper are as follows.

(1) A new PF based fusion model for the real-time vehicle state estimation employing GNSS, electronic compass and road segment.
(2) A new AR based Adaptive high precision vehicle motion model for use with the PF algorithm
(3) Specification and execution of scenarios for simulation and field experiments to demonstrate the superiority of the AR vehicle motion based PF fusion algorithm over GNSS only and PF based fusion with traditional CV and CA vehicle motion models. The performance is measured in terms of the accuracy of the vehicle state estimation and prediction accuracy of potential collision.

The rest of the paper is organized as follows. The fusion algorithm for vehicle real-time state estimation is presented in Section 2. The simulation and field experiments for the evaluation of the proposed algorithm are presented and discussed in Section 3 and Section 4, respectively. The paper is concluded in Section 5.

## 2. Fusion Algorithm-Based V2V Collision Avoidance System

The ability to estimate real-time state of the vehicle is fundamental for collision avoidance and is therefore, an important requirement for the technology chosen. In this case, GNSS based technology with its capability for high accuracy, real-time performance and ease of integration with complementary terrestrial sensors and spatial data, presents a potential solution to deliver state estimation with the required navigation performance (RNP). In this section, the RNP for collision avoidance and the approaches proposed in this paper for state estimation and collision prediction are presented.

### 2.1. Requirement Navigation Performance

Accuracy, integrity, continuity and availability are the main parameters to measure the performance of a navigation system [11,12]. Accuracy refers to the statistical distribution (at the 95th percentile) of position error. Integrity is the ability of a system to provide timely and valid warnings if the position error exceeds a specified alarm limit. Continuity risk is the probability that a service, available at the start of an operation, is interrupted during that operation. Availability measures a navigation system’s operational economy—a service is available if the accuracy, integrity and continuity requirements are satisfied. Before selecting an appropriate navigation system to track vehicle location over time, an assessment of whether the candidate systems satisfy the RNP for collision avoidance systems is required. To date, research has focused on the quantification of the accuracy requirement. The targets for the other parameters (acknowledged to be stringent) are still to be agreed [11]. Therefore, this paper focuses on accuracy and adopts accuracy specified in the SaPPART white paper of 0.5–1 m [12].

### 2.2. Integrated Vehicle State Estimation Framework

**Figure 1:**
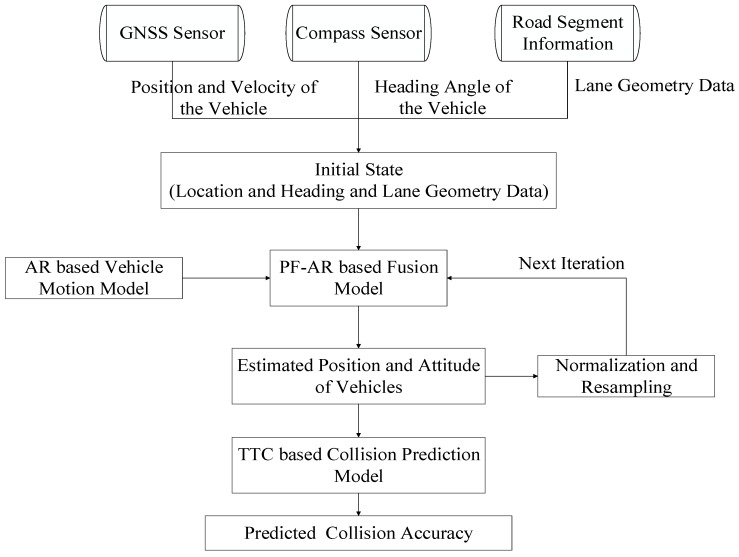
High level processes for the Global Navigation Satellite System (GNSS)/compass/road segment data fusion based vehicle-to-vehicle (V2V) collision avoidance system.

The main processes or steps of the proposed algorithm are presented in Figure 1. Firstly, the collected information from real-time RTK GNSS (positioning and velocity) and electronic compass sensor (heading) for both vehicles as well as the corresponding road segment information (lane geometry data) are used to determine initial state, see Equation (1). The AR based vehicle motion model is then integrated with the vehicle state to feed the PF based fusion algorithm to generate real-time state estimations as described in Section 2.3. The state estimates are then used to predict a potential collision with the Time to Collision (TTC) based collision prediction model and generate the prediction accuracy, as described in Section 2.4.

### 2.3. PF-AR Fusion-Based Vehicle Real-Time State Estimation Model

The vehicle state parameters of time, position and velocity are required for the prediction or detection of a potential collision. Therefore, it is critical to employ the technologies that deliver the RNP in terms of accuracy, integrity, continuity and availability of the relative vehicle states. Table 1 presents the error budget arising from the sensor output and related uncertainties derived from the accuracy requirement for the collision avoidance in [12]. 

The error budget indicates that the required accuracy can be met, assuming that there are no significant GNSS outages that would result in the deterioration in accuracy due to the errors in the electronic compass and spatial data. This is a key aspect of the measurement of the performance for the state estimation algorithm. 

RTK GNSS is based on the principle of differential GPS, which can provide decimeter level positioning accuracy in dynamic mode. A PF based fusion algorithm is designed with the AR motion model to integrate data from RTK GNSS, electronic compass and road segment information with the loosely coupled fusion method. The PF is a type of non-linear filter, which employs a set of weighted samples (particles) to represent a posterior Probability Density Function (PDF), is adaptive to arbitrary distribution and therefore, superior to other non-linear filters, such as EKF, in many state estimation applications [13,14]. In this paper, PF based GNSS/electronic compass/road segment fusion model for horizontal vehicle state estimation is specified as follows.

The defined state vector includes the state from the sensor measurements parameter $\;s=(E_{GNSS}\;N_{GNSS}\;v_{GNSS}\;\theta_{Compass})$ and the state for the road segment parameter $r=(l_{Seg}\;d_{Seg}\;\beta_{Seg}$) for a single vehicle is given by:

(1) $$x={\left[E_{GNSS}\;N_{GNSS}\;v_{GNSS}\;\theta_{Compass}\;l_{Seg}\;d_{Seg}\;\beta_{Seg}\;\right]}^{T}$$

where

➢ $E_{GNSS},\;N_{GNSS}$ are the Easting and Northing coordinates (in meters) of the vehicle’s geometric centre in the local coordinates system;
➢ $v_{GNSS},$ is the heading velocity of the vehicle output from GNSS sensor;
➢ $\theta_{Compass},$ is the heading of the vehicle from compass sensor output;
➢ $l_{Seg}$, is the longitudinal displacement of the vehicle in lane segment coordinates
➢ $d_{Seg}$, is the lateral displacement of the vehicle in lane segment coordinates
➢ $\beta_{Seg}$, is the tangent angle between the tangent line of the lane central line and the Easting-axis coordinates.

**Figure 2:**
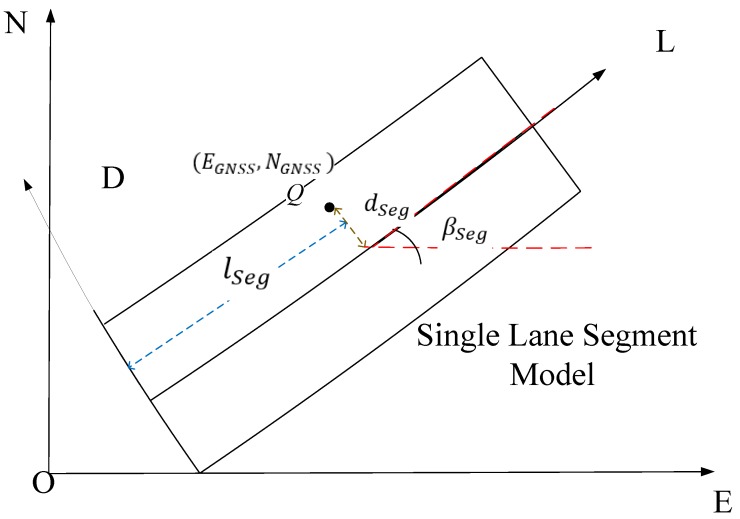
The defined single lane segment model.

The road segment is created based on the road database information. The generation of the lane geometry data for the road segment has been introduced in previous literatures [15,16,17]. One road segment contains several lane segments with lane geometry information. Figure 2 presents the geometric relationships for point *Q* in the single lane segment model. E-N and L-D are the defined local and lane segment coordinate systems, respectively. Assuming that *Q* is the location of the vehicle central point, the corresponding lateral and longitudinal displacements and tangent angle $d_{Seg}\;$, $l_{Seg}$ and $\beta_{Seg}$, respectively. In this paper, we used straight lane segment model, therefore, $\beta_{Seg}$ is a constant value for each lane segment.

The main steps for the PF-AR based fusion algorithm are presented below.

(1) Initialization

The parameters in the PF initialization, are expressed as:

(2) $$x_{t}^{i}\;\left(\;t=0\ldots n;i=1\ldots n\right)$$

where $x_{t}^{i}$, are the parameters in the state vector (1) at the time epoch t with the particle number i.

The filter operation starts with the initialization of the particles $E_{GNSS}{}_{0}^{i},\;N_{GNSS}{}_{0}^{i}$ of the vehicle state vector. The initial coordinates $E_{GNSS}{}_{0}^{i},N_{GNSS}{}_{0}^{i}$ are generated following a Gaussian distribution with the first acceptable GNSS estimation as the mean value with a standard deviation from the *a posteriori* solution statistics. The initial velocity $v_{GNSS}{}_{0}^{i}$ is 0, since at the start the vehicle is assumed to be static. The initial heading is assumed to be along the centre line direction of the road segment, in which the vehicle is located and thus the values for $\theta_{Compass}{}_{0}^{i}$ is set as the corresponding $\beta_{Seg}$. The PF weight assigned to the parameters in the state vector x are noted as $D_{w}\left(x\right),$ determined from the PDF according to expression (21). 

(2) Filter Prediction

A vehicle motion model is required for the prediction stage of the filter. Considering the various maneuvers that could be made when a vehicle is moving, the assumption of a constant motion model is not always valid. Therefore, the traditional CV and CA models, which by definition do not adapt to variations in vehicle motion, could result in excessive errors in vehicle state estimation. 

In this paper, an AR based adaptive motion model is constructed to predict vehicle motion. The AR model is a linear prediction model. Contrary to the traditional motion models, which only use the information of the latest epoch, the AR based model uses current and historical data to better predict the value at a specific future point in time, e.g. point N. The AR model is expressed as:

(3) $$P_{t+1}=\varphi_{1}P_{t}+\varphi_{2}P_{t-1}+\varphi_{3}P_{t-2}+\ldots +\varphi_{p}P_{t-p+1}+a_{t}$$

where $P_{t}$ is the historical data, $a_{t}$ is the noise and $\varphi_{j}$(*j* = 1, 2, …, p) is the regression coefficient.

The estimation of the coefficients in AR model could be achieved as follows.

The matrix of the predicted samples based on their historical data is noted as s in (4).

(4) $$s={\left[P_{t+1}\;P_{t+2}\ldots \;P_{N}\right]}^{T}$$

The matrix containing the noise of the model is noted as *γ* in (5)

(5) $$\gamma ={\left[a_{t+1}\;a_{t+2}\ldots \;a_{N}\right]}^{T}$$

The matrix containing the regression coefficients is noted as φ in (6)

(6) $$\varphi ={\left[\varphi_{1}\;\varphi_{2}\ldots \;\varphi_{p}\right]}^{T}$$

The transition matrix A could be expressed as (7)

(7) $$A=\left[\begin{array}{ccc} \begin{array}{cc} P_{t} & P_{t-1}\\ P_{t+1} & P_{t} \end{array} & \cdots & \begin{array}{c} P_{t-p+1}\\ P_{t-p+2} \end{array}\\ \vdots & \ddots & \vdots \\ \begin{array}{cc} P_{N-1} & P_{N-2} \end{array} & \cdots & P_{N-p} \end{array}\right]$$

Therefore, the AR model could be written as:

(8) s=Aφ+γ

The Least Square (LSQ) solution for the regression coefficient matrix φ, noted as $\hat{\varphi }$, could be calculated as follows.

(9) $$\hat{\varphi }={\left(A^{T}A\right)}^{-1}A^{T}s$$

In our model, the prediction of the s vector is undertaken using Equations (10)–(13). Our research has shown that the most proper number of historical data could be obtained when *t* = 50 (i.e., the previous 50 historical data for the AR model operation), by considering the calculation volume and estimation accuracy. Therefore, we set *t* = 50 for the AR operation in the simulation and filed test.

(10) $$s_{t+1}^{i}=\left[\begin{array}{c} E_{GNSS}{}_{t+1}^{i}\\ \begin{array}{c} N_{GNSS}{}_{t+1}^{i}\\ \begin{array}{c} v_{GNSS}{}_{t+1}^{i}\\ \theta_{Compass}{}_{t+1}^{i} \end{array} \end{array} \end{array}\right]=A_{t}^{i}\varphi_{t}+a_{t}$$

(11) $$A_{t}^{i}=\left[\begin{array}{cccc} E_{GNSS}{}_{t}^{i} & E_{GNSS}{}_{t-1}^{i} & \ldots & E_{GNSS}{}_{t-p+1}^{i}\\ N_{GNSS}{}_{t}^{i} & N_{GNSS}{}_{t-1}^{i} & \ldots & N_{GNSS}{}_{t-p+1}^{i}\\ v_{GNSS}{}_{t}^{i} & v_{GNSS}{}_{t-1}^{i} & \ldots & v_{GNSS}{}_{t-p+1}^{i}\\ \theta_{Compass}{}_{t}^{i} & \theta_{Compass}{}_{t-1}^{i} & \ldots & \theta_{Compass}{}_{t-p+1}^{i} \end{array}\right]$$

where $A_{t}^{i}$ is the matrix for the states from time epoch *t* – *p* + 1 to *t*.

(12) $$\varphi_{t}={\left[\varphi_{X_{t}}\;\varphi_{Y_{t}}\;\varphi_{v_{t}}\;\varphi_{\theta_{t}}\right]}^{T}$$

where $\varphi_{X_{t}},\varphi_{Y_{t}},\varphi_{v_{t}}\;\mathrm{and}\;\varphi_{\theta_{t}}$ are the regression coefficients for $E_{GNSS}{}_{t}^{i}$, $\;N_{GNSS}{}_{t}^{i}$, $v_{GNSS}{}_{t}^{i}$ and $\theta_{Compass}{}_{t}^{i}$.

(13) $$a_{t}={\left[a_{x_{t}}a_{y_{t}}\;a_{v_{t}}\;a_{\theta_{t}}\right]}^{T}$$

where $a_{x_{t}},a_{y_{t}},\;a_{v_{t}},\;a_{\theta_{t}}$ are the random noise for the $E_{GNSS}{}_{t}^{i},$ $N_{GNSS}{}_{t}^{i}$, $v_{GNSS}{}_{t}^{i},\;\theta_{Compass}{}_{t}^{i}$ in the time epoch *t*.

The difference between the states in time epoch *t* + 1 and time epoch *t* is

(14) $$\Delta_{s_{t}}^{i}={\left[\Delta_{X_{{GNSS}_{t}}}^{i}\;,\Delta_{Y_{{GNSS}_{t}}}^{i}\;,\Delta_{v_{{GNSS}_{t}}}^{i},\Delta_{\theta_{{Compass}_{t}}}^{i}\right]}^{T}=s_{t+1}^{i}-s_{t}^{i}$$

Therefore, the prediction for the lane segment related parameters l are expressed as:

(15) $$l_{Seg}{}_{t+1}^{i}=l_{Seg}{}_{t}^{i}+\cos\left(\beta_{Seg}{}_{t}^{i}\right)\Delta_{X_{{GNSS}_{t}}}^{i}+\sin\left(\beta_{Seg}{}_{t}^{i}\right)\Delta_{Y_{{GNSS}_{t}}}^{i}$$

(16) $$d_{Seg}{}_{t+1}^{i}=d_{Seg}{}_{t}^{i}+\sin\left(\beta_{Seg}{}_{t}^{i}\right)\Delta_{X_{{GNSS}_{t}}}^{i}-\cos\left(\beta_{Seg}{}_{t}^{i}\right)\Delta_{Y_{{GNSS}_{t}}}^{i}$$

(17) $$\beta_{Seg}{}_{t+1}^{i}\approx \beta_{Seg}{}_{t}^{i}$$

(3) Weighting and Filter Update

The valid predictions are only based on the valid current particles. Therefore, validity checking is applied only to the current particles. In order to fully use the constraints of the constructed road segment information, the validity of $l_{Seg}{}_{t+1}^{i}$ and $d_{Seg}{}_{t+1}^{i}$ are first checked using Equation (18) to determine if the vehicle is within the same lane segment in a given time interval.

(18) $$\left|l_{Seg}{}_{t+1}^{i}\right|<L\;and\;\left|d_{Seg}{}_{t+1}^{i}\right|<HD$$

where L is the lane segment length and HD is half the length of the width of the lane segment.

If (18) is satisfied, the predictions in (15) and (16) are accepted and the segment number is the same as the previous one. However, if (18) is not satisfied, the two possible cases emerge: (1) the vehicle is moving from the current segment to the next; and (2) the vehicle is not within any lane segment. For the former case, the lane segment is updated, while for the latter case, the particle is considered as invalid and weighted as 0, which also indicates the invalidity of the particle from the previous epoch. 

The weighting and update of the map segment parameter are followed by the sensor measurement parameter. The validity of GNSS position estimates is first checked based on the Receiver Autonomous Integrity Monitoring (RAIM) and if valid, used to adjust the predicted particles.

(19) $$e_{x}^{i}=m_{x_{t+1}}-x_{t+1}^{i}$$

where the $e_{x}^{i}$ is the difference between the real-time measurement vector $m_{x_{t+1}}$ for the sensor measurement parameters and the predicted particles of the parameters in the state vector at epoch t+1. The real-time measurement parameters are generated by the relevant sensors, while the road segment parameters are calculated based on the sensor readings and transformed from the local to the lane segment coordinate system.

As it is known that the normal distribution is parametrized in terms of the mean and the variance, denoted by *μ* and $\sigma^{2}$ respectively, giving the family of densities as follows [18].

(20) $$f\left(x;\mu ,\sigma^{2}\right)=\frac{1}{\sqrt{2\pi }\sigma }exp\left(-\frac{{\left(x-\mu \right)}^{2}}{2\sigma^{2}}\right)$$

We define $D_{w}\left(x_{t+1}^{i}\right)$ as the weight distribution for the particle i for the parameters in the state vector x in the time epoch t+1, σ is the standard deviation of the estimator. The $e_{x}^{i}$ is the difference between the real-time measurement vector $m_{x_{t+1}}$ for the sensor measurement parameters and the predicted particles of the parameters in the state vector at epoch t+1, which is calculated in Equation (19). We substitute Equation (19) to Equation (20) to obtain the Equation (21)

(21) $$w_{t}^{i}=D_{w}\left(x_{t+1}^{i}\;\right)\frac{1}{\sqrt{2\pi }\sigma }\;exp\left(-\frac{\sum^{\text{​}}e_{x}^{i}{}^{2}}{2\sigma^{2}}\right)$$

Finally, the estimated state vector x is calculated by the average of the filter estimated particle values.

For the next iteration, the weights of the particles are modified based on weight distribution$\;D_{w}\left(x_{t+1}^{i}\;\right)$ and normalization and Sequential Importance Resampling (SIR) based resampling will be carried out [19]. 

### 2.4. Collision Avoidance

As stated in [8] potential collisions can be predicted based on the states of proximate vehicles. Therefore, the output of PF-AR estimated vehicle states from Section 2.3 are used as the input to the collision prediction model. In Section 2.4, Time-to-Collision (TTC), which is one of the most common indicators to determine dangerous situations, is introduced. 

**Figure 3:**
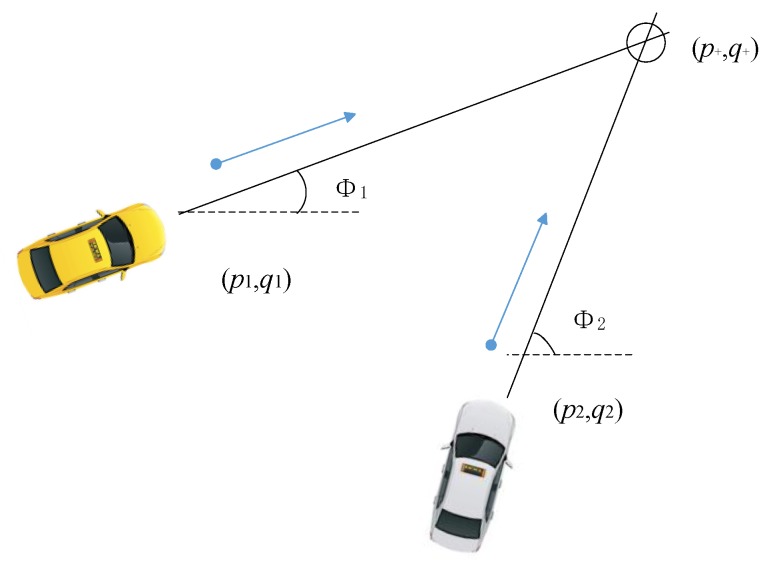
An example of collision prediction.

The definition of TTC is based on the time taken for the two vehicles to collide based on the vehicles’ current relative speed and headings. The procedure to calculate the TTC between two vehicles is presented in [20]. The vehicles states are represented by their known positions, speeds and directions (Figure 3). The calculation of the cross over or intersection point of the two vehicles is, given by the following expressions:

(22) $$p_{+}=\frac{\left(q_{2}-q_{1}\right)-\left(p_{2}tan\left(\emptyset_{2}\right)-p_{1}tan\left(\emptyset_{1}\right)\right)}{tan\left(\emptyset_{1}\right)-tan\left(\emptyset_{2}\right)}$$

(23) $$q_{+}=\frac{\left(p_{2}-p_{1}\right)-\left(q_{2}cot\left(\emptyset_{2}\right)-q_{1}cot\left(\emptyset_{1}\right)\right)}{cot\left(\emptyset_{1}\right)-cot\left(\emptyset_{2}\right)}$$

The position of two vehicles are ($p_{1},q_{1}$) and ($p_{2},q_{2}$). The speeds and directions of the two vehicles are $v_{1},\;v_{2}$ and $\emptyset_{1},\;\emptyset_{2}$ respectively. And the expected intersection point is ($p_{+}$,$\;q_{+}$). Actually, as the vehicles are not just single points, the vehicles will collide before the expected collision point. Note that firstly, the vehicle states refer to a single point on each vehicle (need to consider both size of the vehicle and type of collision) and secondly are assumed in this case to be error-free. Both of these should be accounted for in more sophisticated models.

Once the intersection is computed, the time for each vehicle to arrive at the intersection point is obtained from distance and velocity. The TTC is obtained when the two times are equal [20]. To account for vehicle size and state estimation error, a buffer value ε is introduced in the TTC. The larger value of ε need to be set if the vehicle size or the state estimation error is bigger. The larger value for ε lead to more conservative behaviors by the algorithm. However, if it is too conservative, there will be negative effects, like annoying and desensitizing the driver. Therefore, ε needs to be tuned to achieve the best possible driver experience. The components of TTC are Time-to-Alarm (TTA), the maximum time that elapses after collision detection for the driver to be warned of an impending collision, Driver Reaction Time (DRT) and the Stopping Time (ST) which is the temporal aspect of Stopping Distance (SD) which is related to vehicle’s response time and road conditions. Considering the cases from previous investigations, DRT is usually between 1.5 and 2 s [21]. 

For safe detection of collision, the following condition should be satisfied.


TTA + DRT + ST + *ε* ≤ TTC (24)

Vehicle stopping distances and times are also crucial to determine the time it takes to stop following the application of the brakes. This is related to the vehicle model, road condition and real-time speed at the moment braking is applied. In most of real systems and for simplicity SD is calculated instead of stopping time [22]. Therefore, SD is adopted in this paper and the relationship between the stopping distance and time are calculated in (25). Assuming linearly decreasing speed while braking, then the stopping time can be computed as follows [9]:

(25) $$t=\frac{2S}{v_{0}}$$

where *t* is the stopping time, S is the SD and $v_{0}$ is the pre-braking speed.

## 3. Simulation

The performance of the proposed PF-AR data fusion based collision prediction was initially assessed by simulation. Comparisons are made with traditional PF data fusion algorithms employing the CV and CA vehicle motion models. 

The vehicle reference trajectory and measurements (RTK GNSS positions, electronic compass and road segment data) are simulated in Matlab. The reference vehicle trajectory is first generated by the CA and CV based motion models. After the reference trajectory was generated, various errors were added to the reference to generate the simulated RTK GNSS data and electronic compass data for the desired error budget, see Table 2. In addition, the road segment data were generated using Matlab with the grid accuracy of 0.05–0.1 m. 

Table 3 presents the data for three test cases in an urban environment. From previous data collected in city environments, GNSS outages typically range from 1 to 7 s [23]. Therefore, an outage of 7 s is generated for each test case to investigate if the performance of the designed algorithm meets the accuracy requirement for collision avoidance during GNSS outage. For each test case 1500 collisions were simulated. As all the simulated cases involved collisions, the focus here is therefore, on the ability of the proposed algorithm to detection collisions and not false detection. Furthermore, for each type of collision, a number of velocity scenarios were generated. For example, for rear-end collision, the two scenarios were considered firstly when the leading vehicle A is moving at a constant velocity and collides with the leading vehicle B which is sudden decelerating. The second scenario is when the leading vehicle A is abrupt decelerating while the following vehicle B is abrupt accelerating. A collision was simulated assuming that the distance between the two vehicles is 4.5 m, by considering the general car size. 

The horizontal positioning accuracy for both vehicles from the proposed PF-AR based fusion and the traditional CV and CA based algorithms, are compared with the reference trajectory to determine if the accuracy requirement of 0.5–1 m (95%) can be fulfilled. A summary of the position fixes from PF-AR, PF-CV and PF-CA are shown in Table 4. It is shown that the PF-AR estimated results improve the accuracy of the positioning significantly compared to the CV and CA scenarios in these three test cases. The 95% percentile accuracy in the head-on collision test case is 0.31 m (95%) for the PF-AR algorithm and 1.18 m (95%) and 1.09 (95%) for the CV and CA based scenario. In the intersection perpendicular collision case, the positioning accuracy is 0.30 m (95%) for the PF-AR algorithm, 1.21 m (95%) in the CV scenario and 1.04 m (95%) for CA. In the rear-end collision test case, the positioning accuracy is 0.28 m (95%) for the PF-AR algorithm, 1.13 m (95%) for CV and 0.92 m (95%) for CA. Overall, the PF-AR algorithm has significantly improved the positioning accuracy compared to the other traditional motion model based scenarios.

**Figure 4:**
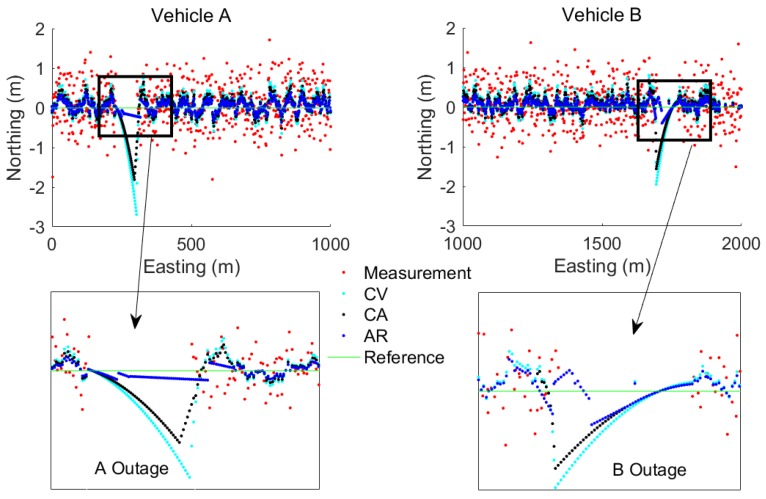
An example of fusion model estimated results for TC1 with head-on collision type.

**Figure 5:**
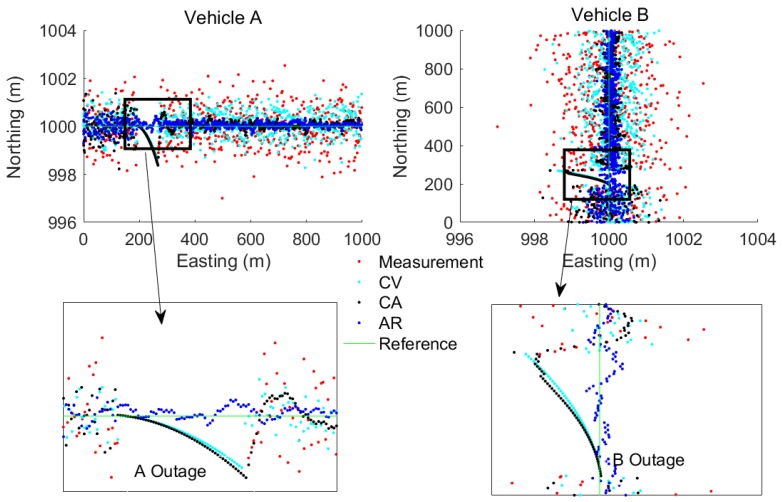
An example of fusion model estimated results for TC2 with intersection perpendicular collision type.

**Figure 6:**
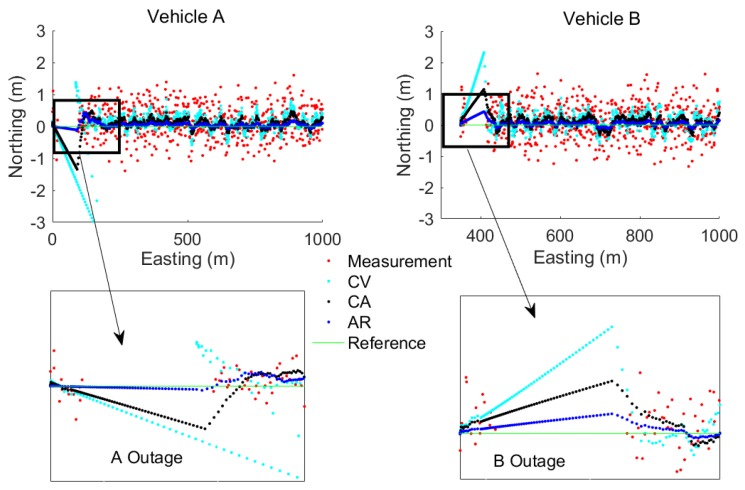
An example of fusion model estimated results for TC3 with rear-end collision type.

Figure 4, Figure 5 and Figure 6 show the benefit of the fusion models compared to the measurements. It is clear that the fusion models could bridge the gaps during the three test cases and that the proposed AR model based fusion has the highest accuracy among the other traditional CV and CA motion model based fusion. It is also noted that, during the GPS outage, the AR based fusion still performs a high accuracy while the CV and CA based fusion models diverge.

**Figure 7:**
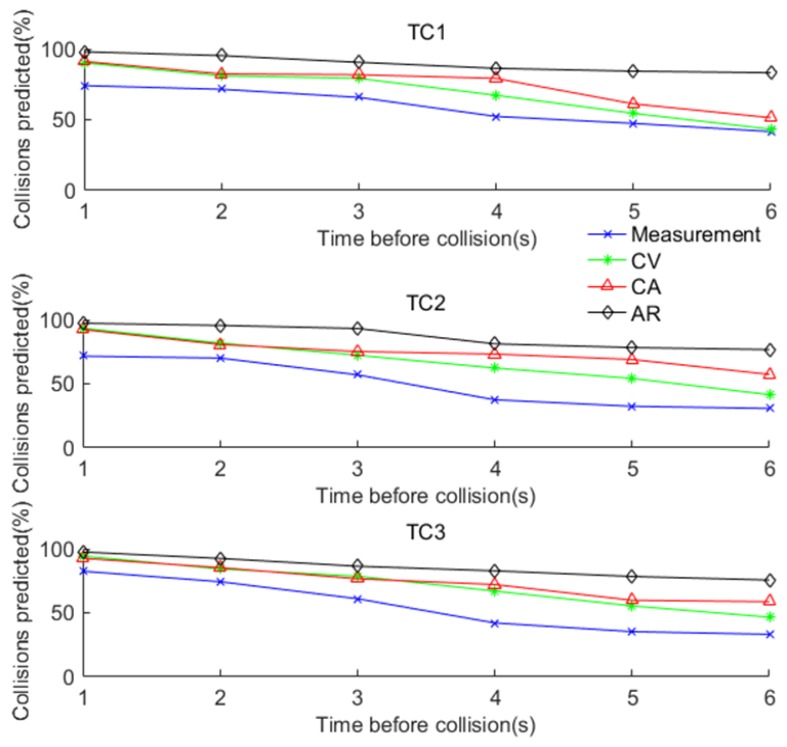
Collision prediction with various positioning methods.

The simulation results of the prediction accuracy based on different positioning algorithms are shown in Figure 7. The percentage of correctly predicted collisions is displayed as a function of time before the collision. It can be seen that the AR based model greatly improves the collision prediction performance, when compared to CV and CA based fusion models. Incidentally, the AR based method is the only one that meets the accuracy requirement 0.5 m (95%). The high positioning accuracy together with the AR based algorithm, which has full use of historical information, results in a relatively high and stable collision avoidance prediction accuracy ranging from 80% (6 s before collision) to 100% (1 s before collision). It should be noted that collision prediction can be determined for any TTC and that a threshold value is required for collision avoidance. This threshold can be determined using Equation (24) based on the values assigned to the TTA and DRT and the calculated values of ST and ε.

## 4. Initial Field Test and Results Analysis

A field experiment was carried out to validate the proposed GNSS/electronic compass/road segment information fusion algorithm for V2V collision avoidance. The test was designed in three stages: (1) equipment set-up and data collection (Section 4.1); (2) comparison of the proposed PF-AR fusion algorithm with other traditional motion model based algorithms for the high-precision vehicle state estimation and assessment of the performance of the algorithms for the prediction accuracy of potential collision (Section 4.2).

### 4.1. Equipment Set-up and Data Collection

**Figure 8:**
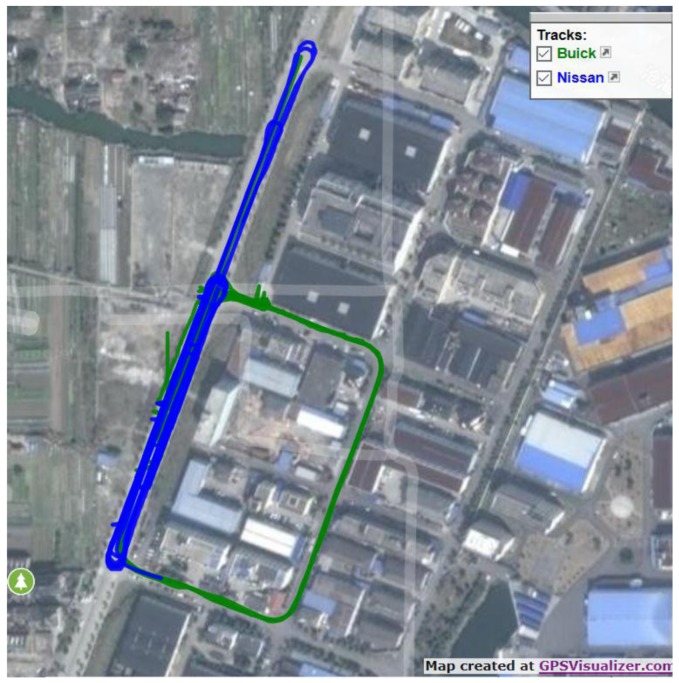
Test trajectories of the two vehicles.

**Figure 9:**
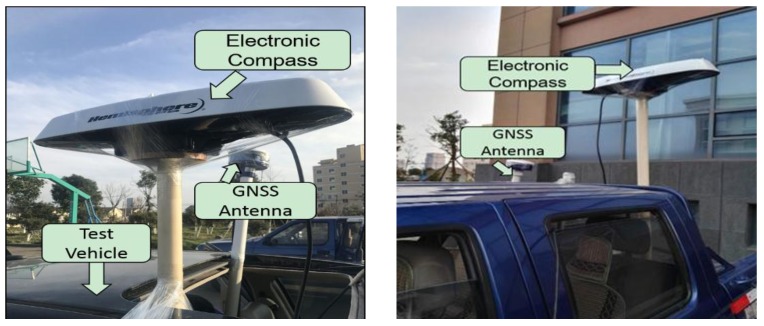
Equipment installation of the two vehicles. Test vehicle 1 (**left**) and Test vehicle 2 (**right**).

**Figure 10:**
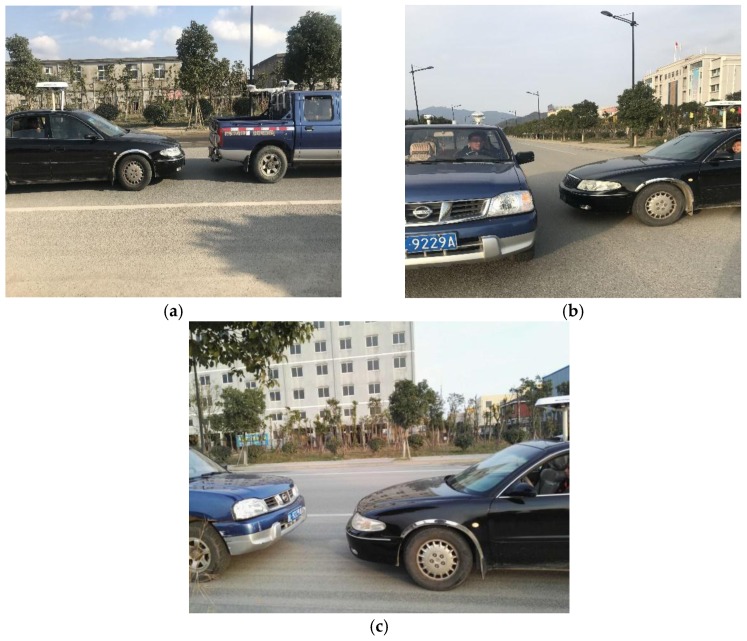
Scenarios designed for the test: (**a**) rear-end collision; (**b**) intersection perpendicular collision; (**c**) head-on collision.

The experimental site used is close to the Lincheng industrial park in Zhoushan City, Zhejiang Province, China. The routes of the two vehicles and the equipment installed are shown in Figure 8 and Figure 9 respectively. The GNSS and electronic compass data were captured from 15:15 to 16:10 in (Beijing Time), for the scenarios rear-end, intersection perpendicular and head-on collision scenarios. The 3 scenarios are shown in Figure 10 and defined in Table 5. For the collision avoidance application, the test vehicles were driven at speeds ranging from 20 km/h to 40 km/h and the data collected at 10 Hz. The data derived from the experiment were:

(1) The reference states (position, velocity and heading) data for both vehicles, post-processed from the on-board RTK GNSS and high grade IMU integration (from I-Mar RT-200) as well as the driving and collision point information recorded by a video;
(2) The RTK GNSS positioning and velocity data for both vehicles, from the ComNav GNSS RTK network and heading of both vehicles from Hemisphere electronic compass.
(3) Road Centreline data, collected by driving a vehicle equipped with an integrated GNSS RTK/high grade IMU, along the road centreline. The data captured were post-processed to extract the reference centreline. Base on the road centreline, the lane centreline and lane segment information could be defined.

**Figure 11:**
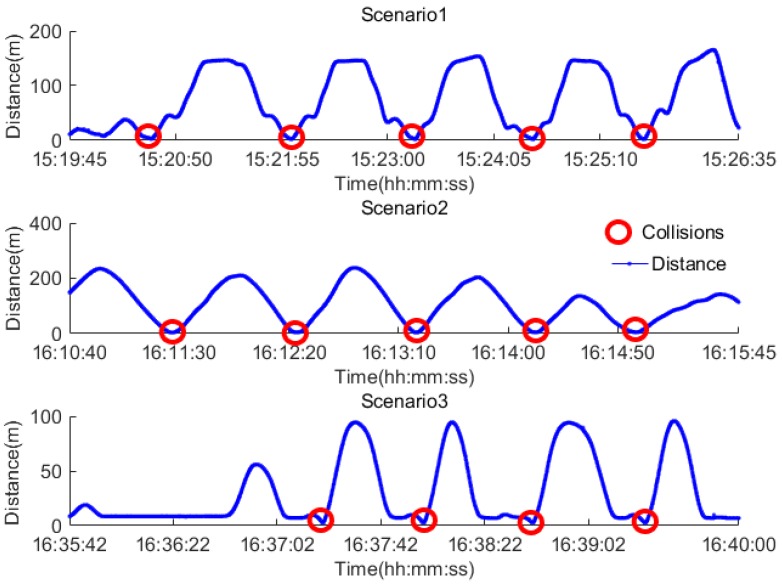
The collision tests.

In order to ensure safety during the experiment, collisions were assumed to occur within a V2V distance of 5 m as shown in Figure 11 by the closest approaches between the two vehicles.

### 4.2. Results Analysis

**Figure 12:**
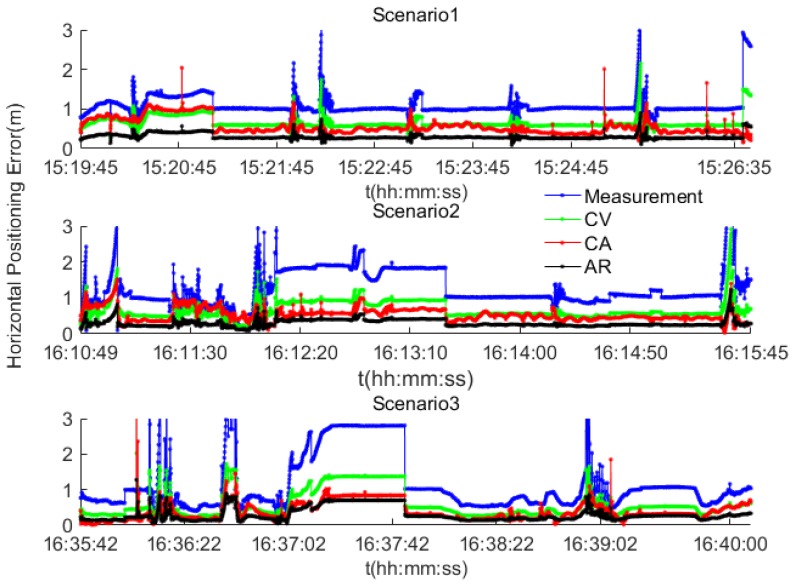
The positioning results for the real tests.

The position errors arising from the use of GNSS only measurements, the AR, CV and CA based data fusion PF algorithms are shown in Figure 12. The 95th percentile accuracy for the route are 0.48 for the PF-AR algorithm, 0.89 m for PF-CA, 1.12 m for PF-CV and 1.48 m for the measurement. Overall, the field test results confirm those from the simulation that the PF-AR algorithm provides the best accuracy and meets the requirement for collision avoidance.

**Figure 13:**
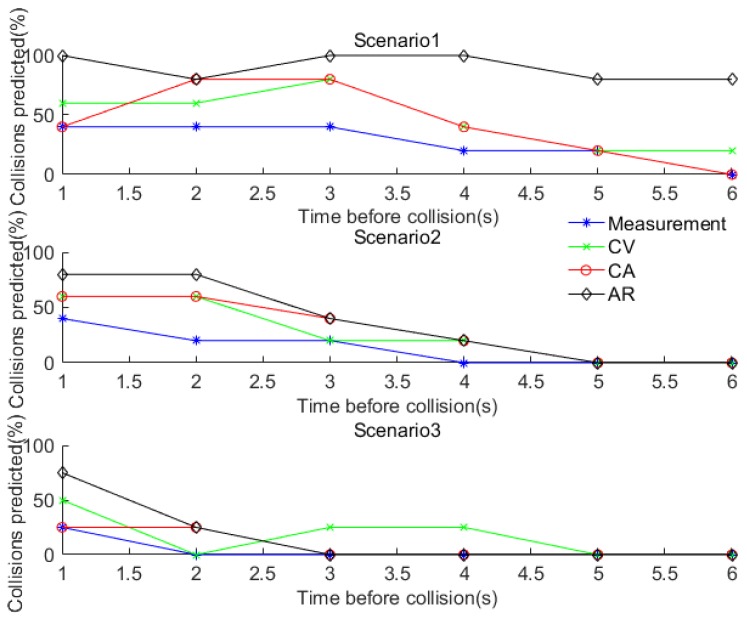
Collision prediction for the field test.

Figure 13 demonstrates how well each navigation method predicts collisions during the field test. In scenario 1, the simulation result is confirmed by the field test results that the AR based fusion algorithm provides comparatively the highest prediction accuracy (e.g., 80%) even at 6 s before the collision, while the other methods have much lower prediction accuracy. However, the prediction accuracy is not good in Scenarios 2 and 3. The reason is that the quality of the RTK GNSS data was poor for these two scenarios due to equipment malfunction and the complex manoeuvres involved. This is an important finding which indicates that the algorithm performance is sensitive to the quality of data and complexity of scenarios including the manoeuvres involved. 

In summary, the GNSS/electronic compass/road information fusion algorithm with PF-AR model has significantly improved positioning accuracy, compared to the other PF fusion algorithms with traditional CV and CA models. Furthermore, the proposed PF-AR model supports highest prediction accuracy than the other algorithms, for example the average prediction accuracy could be 62% for the 2 s and 87% for the 1 s before collisions. Note that a threshold is required to ensure the appropriate TTC that results in collision avoidance. The proposed algorithm can be used to inform the specification of such threshold. 

## 5. Conclusions

This paper presents a novel Particle Filter Autoregressive (PF-AR) motion model based GNSS/electronic compass/road segment information fusion algorithm for the vehicle collision avoidance application. Simulation and field test results have demonstrated the potential of this approach for high accuracy positioning (including in the presence of typical GNSS outages). In comparison to the current algorithms that employ CV and CA models, the proposed PF-AR model meets the 95th percentile horizontal positioning accuracy requirement for the collision avoidance application. 

The proposed algorithm has been shown to not only improve the positioning accuracy and its availability but also the performance of vehicle collision avoidance. The prediction accuracy was analysed as a function of time before the collision. Simulation results show significant improvements both in state estimation accuracy and collision prediction accuracy. The results of simulation are confirmed for scenario 1 by field tests. However, for the more complex scenarios 2 and 3, the low quality of the RTK GNSS data (as a result of equipment malfunction and complexity of manoeuvres) resulted in low performance, pointing to the sensitivity of the algorithm to such issues. Future work will capture and process field data (high density city areas) to investigate these sensitivities and improve the PF-AR model. Furthermore, the other RNP parameters of integrity, continuity and availability will be investigated, following the availability of the agreed standards for collision avoidance. In addition, the communication links between the vehicles and the vehicle 3D navigation performance in complex urban areas will also be considered in the future research. 

## Figures and Tables

**Table 1:** Error budget for the related sources.

Sources	Positioning Error (Standard Deviation, 2*σ*)
RTK GNSS dynamic mode	0.3 m–0.7 m
Electronic heading error	0.1 m–0.3 m
Road segment error	0.05 m–0.1 m
Total positioning error budget	0.32 m–0.77 m

**Table 2:** Simulated trajectory and related noise added.

Simulated Data		Noise	Noise Value Range
RTK GNSS Output	E, N axis coordinates	White Gaussian Noise~N(0, 0.5^2^)	−1.6793~1.3728 m
		Uniformly distributed noise~U(−0.25, 0.25)	−0.2488~0.2500 m
	velocity	White Gaussian Noise~N(0, 0.2^2^)	−0.5228~0.5546 m/s
		Uniformly distributed noise~U(−0.1, 0.1)	−0.0998~0.0999 m/s
Electronic compass	Heading data	White Gaussian Noise~N(0, 0.1^2^)	−0.3154~0.2658 rad
		Uniformly distributed noise~U(−0.05, 0.05)	−0.0497~0.0498 rad

**Table 3:** Simulation test cases.

Test Case (TC)	Data Rate	Number of Samples for Each Vehicle		Collision Type	Gap Duration	Number of Collision
		Vehicle A	Vehicle B			
TC1	10 Hz	667	667	Head-on collision	7 s	1500
TC2	10 Hz	666	666	Intersection perpendicular collision	7 s	1500
TC3	10 Hz	692	692	Rear-end collision	7 s	1500

**Table 4:** The position fixes results for different motion models.

Accuracy Percentage (95%)	Motion Model		
	CV	CA	AR
TC1	1.18	1.09	0.31
TC2	1.21	1.04	0.30
TC3	1.13	0.92	0.28

**Table 5:** Definition of scenarios.

Scenarios	Start Time (Beijing Time)	End Time (Beijing Time)	Collision Type	Number of Collision
1	15:19:45	15:26:35	Rear-end collision	5
2	16:10:40	16:15:45	Intersection perpendicular collision	5
3	16:35:42	16:40:00	Head-on collision	4
